# Pentachlorophenol Induction of the *Pseudomonas aeruginosa mexAB-oprM* Efflux Operon: Involvement of Repressors NalC and MexR and the Antirepressor ArmR

**DOI:** 10.1371/journal.pone.0032684

**Published:** 2012-02-29

**Authors:** Lisa M. Starr, Michael Fruci, Keith Poole

**Affiliations:** Department of Biomedical and Molecular Sciences, Queen's University, Kingston, Ontario, Canada; University of Cambridge, United Kingdom

## Abstract

Pentachlorophenol (PCP) induced expression of the NalC repressor-regulated PA3720-*armR* operon and the MexR repressor-controlled *mexAB-oprM* multidrug efflux operon of *Pseudomonas aeruginosa*. PCP's induction of PA3720-*armR* resulted from its direct modulation of NalC, the repressor's binding to PA3720-*armR* promoter-containing DNA as seen in electromobility shift assays (EMSAs) being obviated in the presence of this agent. The NalC binding site was localized to an inverted repeat (IR) sequence upstream of PA3720-*armR* and overlapping a promoter region whose transcription start site was mapped. While modulation of MexR by the ArmR anti-repressor explains the upregulation of *mexAB-oprM* in *nalC* mutants hyperexpressing PA3720-*armR*, the induction of *mexAB-oprM* expression by PCP is not wholly explainable by PCP induction of PA3720-*armR* and subsequent ArmR modulation of MexR, inasmuch as *armR* deletion mutants still showed PCP-inducible *mexAB-oprM* expression. PCP failed, however, to induce *mexAB-oprM* in a *mexR* deletion strain, indicating that MexR was required for this, although PCP did not modulate MexR binding to *mexAB-oprM* promoter-containing DNA *in vitro*. One possibility is that MexR responds to PCP-generated *in vivo* effector molecules in controlling *mexAB-oprM* expression in response to PCP. PCP is an unlikely effector and substrate for NalC and MexAB-OprM - its impact on NalC binding to the PA3720-*armR* promoter DNA occurred only at high µM levels - suggesting that it mimics an intended phenolic effector/substrate(s). In this regard, plants are an abundant source of phenolic antimicrobial compounds and, so, MexAB-OprM may function to protect *P. aeruginosa* from plant antimicrobials that it encounters in nature.

## Introduction

*Pseudomonas aeruginos*a is an opportunistic pathogen associated with debilitating infections of immunocompromised individuals and individuals with cystic fibrosis [1] and is characterized by an innate resistance to many antimicrobials and the ability to develop/acquire resistance [2]. Significant contributors to this intrinsic and/or acquired multidrug resistance are several members of the RND family of multidrug efflux systems [2]–[4], particularly that encoded by the *mexAB-oprM* efflux operon. MexAB-OprM exhibits one of the broadest substrate profiles of the RND pumps in *P. aeruginosa*, accommodating a wide range of clinically-relevant antimicrobials [5] and biocides [6] as well as a variety of non-clinical agents (e.g., organic solvents [7], [8], dyes [9]–[11], detergents [10], metabolic inhibitors [12], acylhomoserine lactones (AHLs) associated with quorum-sensing (QS) [13], [14]). An important determinant of intrinsic multidrug resistance [9] this efflux system contributes to acquired fluoroquinolone [15]–[17] and β-lactam [18]–[23] resistance in clinical isolates of *P. aeruginosa*.

Expression of the *mexAB-oprM* operon is regulated by the product of a local repressor gene, *mexR* [24], occurring upstream of the efflux genes and the target of mutation in MexAB-OprM-overexpressing multidrug-resistant *nalB* lab [24], [25] and clinical [26]–[28] isolates. MexR acts to control *mexAB-oprM* expression from one of two promoters (PI; most distal from the efflux genes) for this efflux operon [29], [30], binding as a dimer [31] to a site that also overlaps the *mexR* promoter [29], [32] and, so, effects negative autoregulation. Recent studies show that its ability to bind and so repress *mexAB-oprM* is governed by the redox status of the protein, MexR serving to regulate *mexAB-oprM* expression in responsive to oxidative stress [33], [34]. MexR repressor activity is also modulated by the product of the *armR* anti-MexR repressor gene, which binds to MexR and negatively impacts its ability to bind to the *mexAB-oprM* PI promoter region [35], [36]. *armR* occurs as part of the 2-gene operon PA3720-*armR* that is regulated by the product of the divergently-transcribed *nalC* repressor gene [37], with *nalC* mutants showing elevated PA3720-*armR* expression and, so, elevated *mexAB-oprM* expression and multidrug resistance [37] as a result of ArmR modulation of MexR's repressor activity [35]. *nalC* lab and clinical isolates expressing *mexAB-oprM* and showing a multidrug-resistant phenotype have been reported [24], [37], [38]. A third repressor involved in regulating *mexAB-oprM* expression, NalD, is encoded by a gene unlinked to *mexAB-oprM* and *nalC*/PA3720-*armR* and regulates *mexAB-oprM* from a second, efflux operon-proximal promoter (PII) [30]. Mutations in *nalD* have been described in lab and clinical multidrug-resistant mutants [39].

Despite their contribution to antimicrobial resistance, RND family multidrug efflux systems in *P. aeruginosa* are increasingly appreciated as having other than drug efflux as an intended function, most of these systems being regulated independently of antimicrobials and instead induced in response to environmental stress (e.g, MexCD-OprJ, envelope stress [40]; MexEF-OprN, nitrosative stress [41]; MexXY-OprM, oxidative [42] and ribosome [43], [44] stress). Recently, the *mexAB-oprM* efflux system and its regulatory locus *nalC*/PA3720-*armR* have been shown to be inducible by the uncoupler of oxidative phosphorylation and environmental contaminant, pentachlorophenol (PCP) [45], the implication being that this compound is being ‘sensed’ by NalC and *mexAB-oprM* recruited as a result of ArmR-mediated MexR modulation. While MexAB-OprM appears able to accommodate this toxin (mutants lacking the pump are more sensitive to PCP) [45], it is unclear whether PCP is the actual signal/substrate or whether downstream effects of the uncoupling of oxidative phosphorylation are responsible for *nalC*/PA3720-*armR* and *mexR/mexAB-oprM* upregulation. The current study was undertaken to address this and to ascertain the mechanism by which PCP ultimately induces *mexAB-oprM*. We report here that NalC is a PCP-responsive repressor that mediates PCP induction of PA3720-*armR* and, possibly, *mexAB-oprM* but that PCP is also able to induce *mexAB-oprM* expression independently of PA3720-*armR*, possibly as a result of PCP-generated oxidative stress and, so, redox regulation of MexR. During the course of this work another study demonstrating PCP control of NalC repressor activity was published [46] although the mechanism(s) by which this toxin induced *mexAB-oprM* were not assessed, and the assumption that it was via ArmR are not supported by our study.

## Materials and Methods

### Bacterial strains and plasmids

The bacterial strains and plasmids used in this study are listed in Table 1. Bacterial cells were cultured in Luria broth (L broth) and on Luria agar with antibiotics, as necessary, at 37°C. Plasmid pET23a and its derivatives were maintained in *E. coli* with ampicillin (100 µg/ml). Plasmid pEX18Tc and its derivatives were maintained in *E. coli* with tetracycline (10 µg/ml). Plasmid pLysS was maintained in *E. coli* with chloramphenicol (30 µg/ml). ΔPA3720 derivatives of *P. aeruginosa* strains K767 and K3145 (Δ*armR*) were generated by constructing a PA3720 deletion in plasmid pEX18Tc and mobilizing it into these strains as before [47]. Briefly, 1 kb fragments corresponding to sequences 5′ and 3′ to the PA3720 sequences being deleted were amplified using the polymerase chain reaction (PCR) and primers 3720 UF (5′-GCTAGAATTCGTAGGTGGTGAAGCCGAGC-3′; *EcoRI* site underlined) and 3720 UR (5′-GCTAGGTACCTGACGCCGCCATGTCCCT-3′; *KpnI* site underlined), and 3720 DF (5′-GCTAGGTACCCATGGGGGGACTCCTGCG-3′; *KpnI* site underlined) and 3720 DR (5′-GCTAAAGCTTCCTGGTCCTGGCGACACG-3′; *HindIII* site underlined), respectively. The 50 µl PCR reaction mixture contained 1 µg of chromosomal *P. aeruginosa* K767 DNA as template, 1 µM of each primer, 0.3 mM each deoxynucleoside triphosphate (dNTP), 1× Exact buffer, 1× 5P buffer and 2.5 U Exact DNA polymerase (5 PRIME, Inter Medico, Markham, Ontario). Following an initial denaturation step at 95°C for 5 min, the mixture was subjected to 30 cycles of heating at 94°C for 45 sec, 63°C (annealing temperature) for 30 sec, and 72°C for 1 min, before finishing with a10-min incubation at 72°C. PCR products were gel purified, digested appropriately and cloned sequentially into pEX18Tc, to yield the ΔPA3720 vector, pLMS2. Plasmid pLMS2 was mobilized into *P. aeruginosa* K767 from into *E. coli* S17-1 and *P. aeruginosa* transconjugants harbouring chromosomal inserts of the deletion vector were selected on L-agar plates containing tetracycline (50 µg/ml) and chloramphenicol (5 µg/ml; to counterselect *E. coli* S17-1). These were subsequently streaked onto L agar containing sucrose (10% [wt/vol]) as before [48], with sucrose-resistant colonies screened for the appropriate deletion using colony PCR [49] with primers 3720 UF and 3720 DR and parameters detailed above (except for an annealing temperature of 55°C). A Δ*armR* derivative of *P. aeruginosa* K767 was constructed using pEX18Tc::Δ*armR* plasmid pLC8 as described previously [37] except that transconjugants carrying chromosomal inserts of the deletion vector were selected on L-agar plates containing tetracycline and chloramphenicol as above. Δ*armR* mutants were verified by colony PCR [49] using primers 3719UF (5′-CGGTGAGCGTGGCGCCG-3′) and 3720DR (5′-TGGGCTCTTTACCTCAAGT-3′). Reaction mixtures were heated to 95°C for 5 min followed by 30 cycles of 30 sec at 94°C, 30 sec at 60°C, 1 min at 72°C and a final 5-min elongation at 72°C.

**Table 1:** Bacterial strains and plasmids.

Strain	Relevant characteristic	Reference or source
***E. coli***		
DH5α	φ80Δ *lacZ*ΔM15 *endA1 recA1 hsdR17* (r_K_^−^ m_K_^+^) *supE44 thi-1 gyrA96 relA1* F^−^ Δ(*lacZYA-argF)U169*	[59]
K113	BL21 (DE3) (pLysS)	[60]
K114	BL21 (DE3) (pLysE)	[60]
S17-1	*thi pro hsdR recA* Tra+	[61]
Sm10(λpir)	*thi-1 thr lec tonA lacY supE recA::RP4-2-Tc::Mu*; Km^r^ *λpir*	[62]
NovaBlue	*recA^−^ endA^−^ lacI^q^*	Novagen
***P. aeruginosa***		
K767	PAO1 prototroph	[63]
K1454	Spontaneous *nalC* mutant of K767	[24]
K2276	K1454 Δ*armR*	[37]
K2568	K767 Δ*mexR*	[30]
K3145	K767 Δ*armR*	This study
K3146	K767 ΔPA3720	This study
K3130	K767 ΔPA3720-Δ*armR*	This study
K3151	K1454 ΔPA3720	This study
**Plasmids**		
pET23a	His-tag expression vector: Ap^r^	Novagen
pKLE1	pET23a::*mexR*	[29]
pLMS3	pET23a::*nalC*	This study
pEX18Tc	Broad-host-range gene replacement vector; *sacB*; Tc^r^	[64]
pLC8	pEX18Tc::Δ*armR*	[37]
pLMS2	pEX18Tc::ΔPA3720	This study
pMF1	pEX18Tc::ΔPA3720-Δ*armR*	This study

Ap^r^, ampicillin resistant; Km^r^, kanamycin resistant; Tc^r^, tetracycline resistant.

### DNA methods

Standard protocols were used for restriction endonuclease digestion, ligation, transformation, plasmid isolation, agarose gel electrophoresis, and preparation of chemically competent (CaCl_2_) *E. coli* cells as described by Sambrook and Russell [50]. Electrocompetent *P. aeruginosa* cells were prepared as described by Choi and Shwiezer [51]. Chromosomal DNA was extracted from *P. aeruginosa* using the DNeasy Tissue Kit (Qiagen, Inc., Mississauga, Ontario). Plasmid DNAs were also prepared from *E. coli* or *P. aeruginosa* using a GeneJET Plasmid Miniprep kit (Fermentas Canada Inc., Burlington, Ontario, Canada) according to a protocol provided by the manufacturer. The Wizard® SV Gel and PCR Clean-Up System kit (Promega Corp., Nepean, Ontario) was used to purify PCR products and to gel-purify DNA fragments generated by restriction endonuclease digestion. Oligonucleotides were synthesized by Integrated DNA Technologies (IDT, Coralville, Iowa, USA) and nucleotide sequencing was performed by AGCT Corp. (Toronto, Ontario).

### Susceptibility testing

The antimicrobial susceptibilities of the various *P. aeruginosa* strains were assessed in 96-well microtiter plates using twofold serial dilutions as described [52]. In assessing the impact of PCP exposure on antimicrobial susceptibility, 0.75 mM PCP (one quarter of the MIC) was included in the growth medium used to prepare the bacterial inoculum and to generate the serial dilutions.

### Quantitative RT-PCR

Overnight cultures of *P. aeruginosa* strains were subcultured (1∶49) in fresh L-broth and incubated at 37°C with shaking for 2.5 hours, at which time cells were harvested by centrifugation. In some experiments, PCP (0.75 mM final concentration; ¼ MIC) was added 1 to 1.5 hr before harvesting. Total bacterial RNA was isolated from 1 ml of late-log phase culture using the RiboPure™ Bacteria kit (Ambion, Inc., Streetsville, Ontario) or the High Pure RNA Isolation Kit (Roche Diagnostics) using the manufacturers' protocols, and resuspended in 50 µl elution buffer. Samples were treated with Turbo DNA-Free (Ambion, Inc; 2 U enzyme per 10 µg of RNA for 60 min at 37°). DNA-free RNA (confirmed usng PCR; 1 µg) was used to synthesize cDNA using an iScript cDNA Synthesis kit (Bio-Rad, Mississauga, Ontario) in a reaction mixture formulated as described by the kit manufacturer and incubated for 5 min at 25°C, 30 min at 42°C and 5 min at 85°C. cDNA was then stored until needed at −20°C. Quantification of the cDNA was carried out using a Bio-Rad, CFX96™ Real-Time PCR Detection System (Bio-Rad, Mississauga, Ontario). PCR amplification reactions were performed in 20 µl reaction volumes each containing 10 µl of SsoFast EvaGreen Supermix (Bio-Rad, Mississauga, Ontario), 0.6 µM each of 2 primers per gene being amplified (*mexA*: mexAQ-F, 5′-CAGCAGCTCTACCAGATCG-3′ and mexAQ-R, 5′-CGTACTGCTGCTTGCTCA-3′; PA3720: 3720Q-F, 5′-GATGCCTTTCCCTTGGTC-CA-3′ and 3720Q-R, 5′-TCCTTGAGCCACAACACCAG-3′; *rpsL*: rpsLQ-F, 5′-GGT-TTCCTCGTACATCGGTG-3′ and rpslQ-R, 5′-TGCTTACGGTCTTTGACACC-3′) and 5 µl of 1∶49 diluted cDNA. Following an initial 3-min denaturation at 95°C, the mixture was subjected to 40 cycles of 10 sec at 95°C and 30 sec at 60°C. A melt curve, obtained following an initial 10-sec treatment at 95°C and involving 5-sec incubations of 0.5°C increments beginning at 65°C, was run at the end of 40 cycles to test for the presence of a unique PCR product. The expression levels of *mexA* and PA3720 were normalized to that of the reference gene *rpsL*. Biological duplicates and technical triplicates were performed for all samples. To confirm the absence of genomic DNA contamination, ‘no-template’ controls were performed in technical triplicates for all primer sets employed.

### Expression and purification of polyhistidine (His)-tagged NalC protein

The *nalC* gene was amplified by PCR using primers: nalC-Fwd-NdeI (5′-GATCCATATGAACGATGCTTCTCCCCGTCTG-3; NdeI site underlined) and nalC-Rev-SalI (5′-GATCGTCGACGCCCTGCGCGGGGCTCTG-3′; SalI site underlined) in a 50 µl PCR reaction that contained 1 µg *P. aeruginosa* strain K767 chromosomal DNA, 0.2 mM each dNTP, 3% (vol/vol) DMSO, 1× Phusion® High Fidelity (HF) buffer and 1 U Phusion® polymerase (Finnzymes, New England Biolabs, Pickering, Ontario). The reaction mixture was heated for 30 sec at 94°C followed by 30 cycles of 30 sec at 94°C, 30 sec at 54°C and 30 sec at 72°C, before finishing with 10 min at 72°C. The *nalC*-containing PCR product was purified, digested with SalI and NdeI and cloned into NdeI-XhoI-restricted pET23a (Novagen, Madison, Wisconsin, USA) to yield pLMS3 encoding His-tagged NalC (i.e, NalC-His). Following nucleotide sequencing of the cloned gene to confirm the absence of PCR-generated mutations, plasmid pLMS3 was introduced into *E. coli* BL21 (DE3) carrying the pLysS plasmid and NalC-His production induced with IPTG using a modified version of a previously-described protocol [29]. Briefly, an overnight culture of *E. coli* BL21 (DE3) carrying pLysS and pLMS3 was diluted 1∶49 in LB (50 ml) supplemented with the appropriate antibiotics and incubated at 37°C until the optical density at 600 nm reached 0.5–0.6, at which time IPTG (1 mM final concentration) was added and incubation continued a further 2 hr. Cells were harvested by centrifugation (10 min at 10000×g) at 4°C and pellets were resuspended in 6 ml buffer A (0.3 M NaCl, 50 mM Na_2_HPO_4_) containing 5 mM imidazole and sonicated (three sonic bursts of 30 sec, power 40 with a VibraCell Sonicator [Sonics & Material Inc., Danbury, Connecticut, USA]). Following centrifugation for 60 min at 16000×g, the NalC-His-containing supernatant was mixed with 500 µl of Ni-NTA Agarose resin (Qiagen, Inc.) equilibrated with 10 ml buffer A containing 5 mM imidazole and incubated, with shaking, for 10 min. The resin was subsequently pelleted by centrifugation (3 min at 3000×g) and washed twice with 10 ml buffer A containing 5 mM imidazole and once with 2 ml buffer A containing 5 mM imidazole, the resin again being centrifuged after each wash. Bound protein was eluted stepwise with 500 µl buffer A containing increasing amounts of imidazole (50, 100, 150, 250 mM); at each step the resin was incubated with shaking at room temperature and centrifuged as above. The NalC-His protein was recovered in the supernatant following elution with buffer A containing 250 mM imidazole. Protein concentration was determined using the BCA Protein Assay Kit (Pierce, Illinois, USA), and purified protein (verified using SDS-polyacrylamide gel electrophoresis [10]) was stored at −20°C in 20% (vol/vol) glycerol.

### Electromobility shift assay

The binding of purified NalC (see above) and MexR (prepared as above but using pET23a::mexR vector pKLE1 in place of pLMS3) to PCR-amplified target DNAs was assessed using the electromobility shift assay (EMSA) as described previously [30]. Briefly, 50 ng target DNA was incubated with purified NalC or MexR for 20 min at room temperature in a 15 µl reaction mixture containing 1× binding buffer (750 mM KCl, 0.5 mM dithiothreitol, 0.5 mM EDTA, 50 mM Tris-HCl, pH 7.4). Following the addition of EMSA gel-loading solution, mixtures were separated by electrophoresis on a non-denaturing 8% (wt/vol) polyacrylamide gel in 0.5 TBE buffer (22 mM Tris-HCl, 22 mM boric acid, 0.5 mM EDTA, pH 8.0) and gels were stained with 1× SYBR Green EMSA nucleic acid stain. DNA was then visualized using digital photography with a S6656 SYPRO photographic filter (S6656).

NalC target DNAs included the entire nalC-PA3720 intergenic region (205 bp), ca. 100 bp fragments corresponding to the PA3720-distal and -proximal halves of this region as well as shorter oligonucleotides corresponding to the putative NalC binding site. Amplification of the 205 bp fragment was achieved using primers nalC-3720 Fwd 1 (5′-TGGGGGGACTCCTGCGGG-3′) and nalC-3720 Rev 2 (5′-GAGCGGTATCGGGCCTCG-3′) in a reaction mixture (50 µl) containing 1 µg chromosomal P. aeruginosa K767 DNA, 0.2 mM of each dNTP, 30 pmol of each primer, 1× ThermoPol buffer, 10% (vol/vol) DMSO and 2 U Taq DNA polymerase (New England Biolabs). The mixture was heated at 94°C for 30 sec, followed by 30 cycles of 30 sec at 94°C, 30 sec at 63°C, and 30 sec at 72°C, before finishing with 10 min at 72°C. The 102-bp PA3720-proximal fragment was amplified using primers nalC-3720 Rev 2 and nalC-3720 9 Fwd (5′-CTAACGAGAAACGCTC-3′) in a mixture formulated as above and heated at 94°C for 45 sec, followed by 30 cycles of 45 sec at 94°C, 45 sec at 50°C, and 45 sec at 72°C for 45 sec, again finishing with 10 min at 72°C. The 107-bp PA3720-distal fragment was amplified using primers nalC-3720 Fwd 1 and nalC-3720 8 Rev (5′-TTAGGGTTGACGCTGGTCA-3′) in a reaction mixture containing 1 µg of chromosomal DNA, 0.3 mM each dNTP, 1 µM of each primer, 1× Exact buffer and 2.5 U Exact DNA polymerase (5 PRIME, Inter Medico, Markham, Ontario). The mixture was heated at 95°C for 5 min followed by 30 cycles of 30 sec at 94°C, 30 sec at 65°C, and 1 min at 72°C, before finishing with 7 min at 72°C. Oligonucleotides (41 bp) corresponding to various regions upstream of PA3720-armR (NalC-1, 5′-TGGTCATTT-AAGAACTGTATCGTACAGTACTGTTTTGGCAA-3′; NalC-2, TTGGCAAGCACT-TCCGCTCATTCCTCCGCCTTGCCTCCCGC-3′; NalC-3, TGGTCATTTATCTTGA-CAATCGTACAGTACTGTTTTGGCAA-3′), including a putative NalC-binding site, and their reverse complements were annealed and also used in EMSAs. Briefly, 75 pmol of each oligonucleotide pair was annealed in 50 µl dH_2_0 by heating for 3 min at 95°C, followed by 10 min at 90°C, and 5 min at 10°C decrements to 30°C at which time the annealed oligonucleotides were cooled and stored at 4°C.

The MexR target DNA, corresponding to the 351-bp mexR-mexA intergenic region, was amplified using the primers K9 (5′-CTGAAGATCTGTTGCATAGCGTTG-TCCTCA-3′) and K10 (5′-ACGGGGTACCCGGGGTAGTTCATTGGTTTG-3′) in a reaction mixture formulated as above for the 205-bp nalC-PA3720 intergenic region, except that DMSO was excluded and MgCl_2_ was included at 1.5 mM final concentration. The reaction was heated at 94°C for 5 min, followed by 30 cycles of 1 min at 94°C, 2 min at 56°C, and 1.5 min at 72°C, before finishing with 30 min at 72°C. To assess the specificity of any binding observed, excess salmon sperm DNA (100 µM) was added to the reaction mixtures prior to the addition of protein. In experiments where various compounds or plant extracts were being tested for an ability to interfere with protein-DNA binding, these were also added (concentrations indicated in the appropriate figure legends) prior to adding the protein. Since preparation of PCP required the use of a solvent (10 mM NaOH), control experiments involving 10 mM NaOH only were carried out to ensure that the solvent itself was not impacting the EMSA.

### Mapping the PA3720-*armR* transcription start site

To identify the transcription start site for the PA3720-*armR* operon, the 5′ rapid amplification of cDNA ends (RACE) protocol [53] and a 5′/3′ RACE Kit, 2nd Generation (Roche Diagnostics) were used as described previously with modifications [29]. Total RNA was prepared from PA3720-*armR*-expressing *P. aeruginosa nalC* mutant K1454 using the Roche High Pure RNA Isolation Kit according to the manufacturer's instructions, and contaminating DNA was removed using the Turbo DNase Kit (Ambion). cDNA was synthesized from total RNA (1 µg) with a RACE kit-provided reverse transcriptase and a PA3720-specific primer, 3720 RACE Sp1 (5′-GGCGGATAGACCAGGG CGAAAT-3′; anneals 131 bp downstream of the PA3720 ATG start site), using a protocol provided by the manufacturer. A homopolymeric A-tail was added to the 3′ end of the total cDNA using terminal transferase and dATP, and the dA-tailed cDNA subsequently PCR amplified using a kit-provided oligo (dT)-anchor primer and a second PA3720-specific primer, 3720 RACE Sp2 (5′-CGGCCAGCTCGCGGGGCATCT-3′; anneals 57 bp downstream of the PA3720 ATG start site). The resulting PCR product was purified and cloned into the pETBlue-1 AccepTor™ Vector (EMD Chemicals, Gibbstown, New Jersey, USA) as per instructions provided by the manufacturer. Following transformation of NovaBlue Giga Singles Competent Cells (Novagen), plasmid-carrying *E. coli* cells were selected on L-agar kanamycin (50 µg/ml) and tetracycline (15 µg/ml). Plasmids carrying inserts of the amplified 5′ end of PA3720 mRNA were identified and the inserts sequenced and aligned with the PAO1 genome sequence to determine the PA3720-*armR* transcription start site.

## Results

### NalC mediates PCP inducibility of PA3720-*armR*

**Figure 1:**
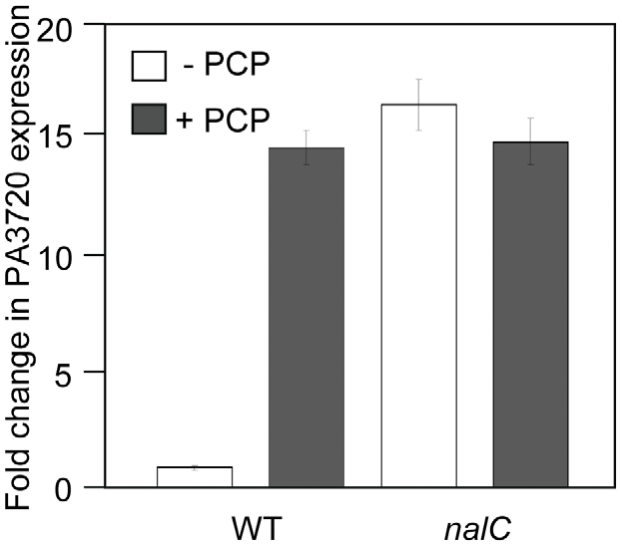
PCP induction of PA3720-*armR*. qRT-PCR results showing impact of PCP and/or a *nalC* mutation on PA3720 expression (as a measure of PA3720-*armR* expression). Expression of PA3720 is reported relative to the *rpsL* internal control for wild type *P. aeruginosa* K767 and its *nalC* derivative, K1454 exposed or not to PCP (0.75 mM; 1.5 hr). Results shown are the mean +/− standard error of one cDNA sample for each, processed in triplicate, and are representative of 2 independent experiments.

**Figure 2:**
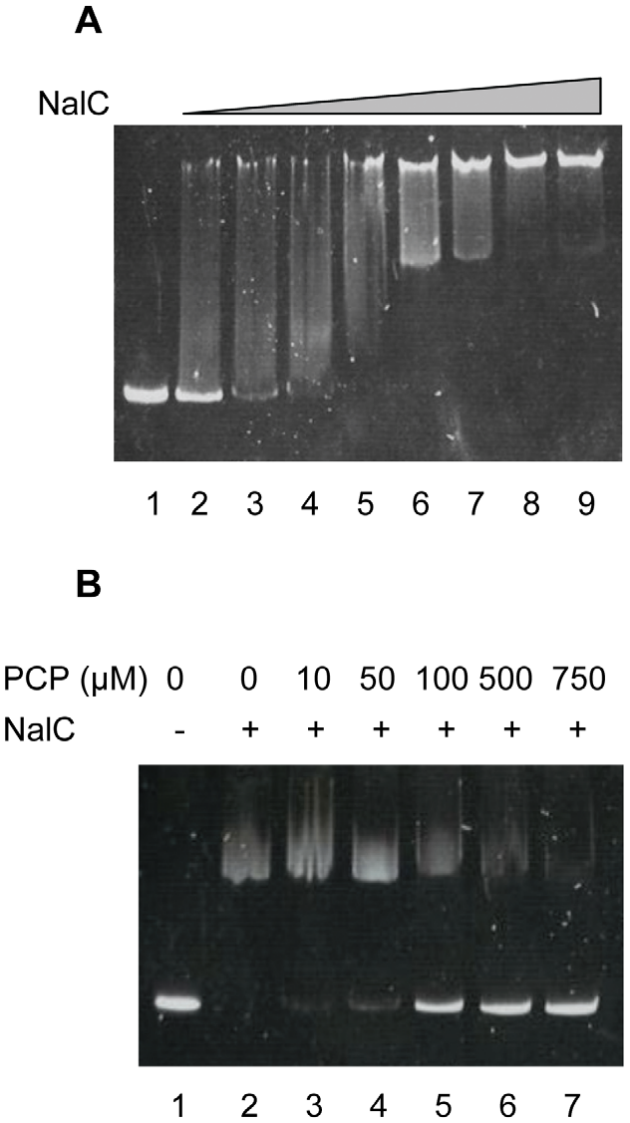
PCP modulation of NalC repressor binding to the PA3720-*armR* upstream region. A) Mobility shift assay in which 50 ng of a 209-bp DNA fragment encompassing the *nalC*-PA3720 intergenic region was incubated without (lane 1) or with 200 (lane 2), 300 (lane 3), 400 (lane 4), 500 (lane 5), 800 (lane 6), 1000 (lane 7), 2000 (lane 8) or 3000 (lane 9) ng of purified NalC-His. B) Mobility shift assay in which purified NalC-His (800 ng) was incubated with 50 ng of a 209-bp DNA fragment encompassing the *nalC*-PA3720 intergenic region and increasing amounts of PCP as indicated. Lane 1, DNA only control.

Microarray data has shown that PCP induces expression of the NalC-regulated PA3720-*armR* operon [45], results that have been confirmed here using quantitative RT-PCR (qRT-PCR) (Fig. 1). PCP induction of PA3720-*armR* was lost in a *nalC* mutant (K1454) – although the mutant showed the expected increase in PA3720-*armR* expression compared with its wild type parent K767, PCP treatment did not enhance expression of this operon in K1454 (Fig. 1). This indicated that NalC was mediating the PCP induction of PA3720-*armR*, either via a direct response to PCP or via recognition of PCP-generated cellular products (PCP is an energy inhibitor). To assess this NalC was purified and the impact of PCP on its binding to the ca. 200 bp *nalC*-PA3720 intergenic region was examined using EMSA. As seen in Fig. 2, NalC bound the *nalC*-PA3720 intergenic region (Fig. 2A) with binding compromised by the addition of PCP (some loss of binding was detected at 10 µM PCP with almost complete loss of binding seen at 750 µM) (Fig. 2B). Thus, NalC responds directly to PCP in mediating its induction of PA3720-*armR*.

### Mapping the NalC binding site

**Figure 3:**
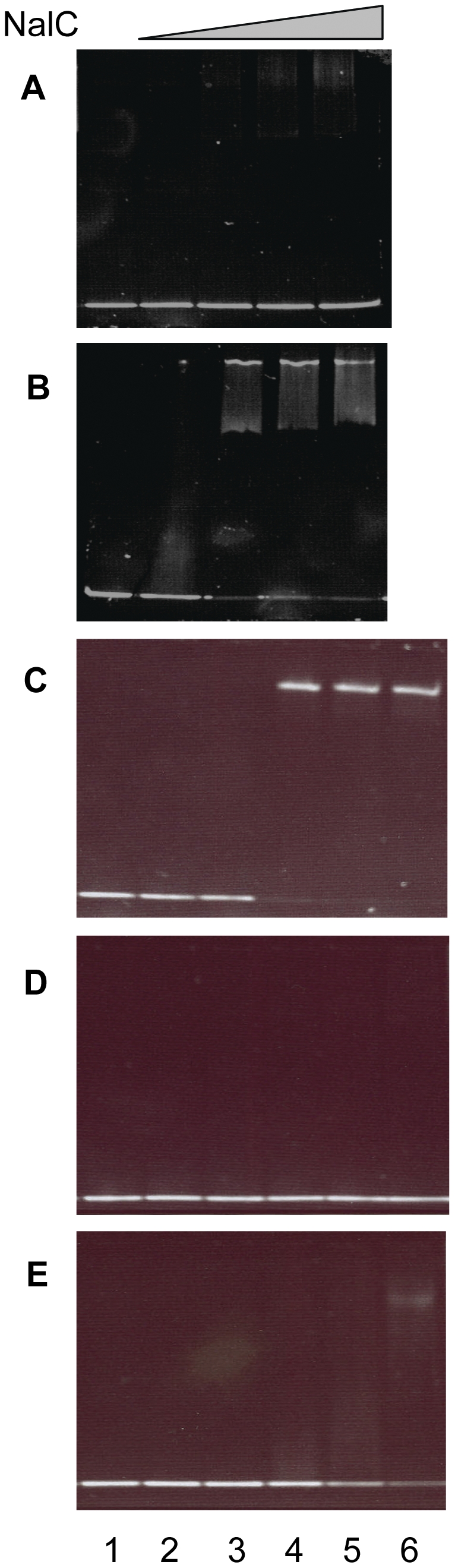
Identifying the NalC binding site upstream of PA3720-*armR*. A–B) Mobility shift assay in which 50 ng of A) the 107-bp PA3720-distal DNA fragment (sequence delineated by endpoints I in Fig. 4A) or B) the 102-bp PA3720-proximal DNA fragment (sequence delineated by endpoints II in Fig. 4A) were incubated without NalC (lane 1) or with 300 (lane 2), 500 (lane 3), 800 (lane 4) or 1000 (lane 5) ng of purified NalC-His. C–E) Mobility shift assay in which 0 (lane 1), 300 (lane 2), 500 (lane 3), 800 (lane 4) and 1000 (lanes 5) and 2000 (lanes 6) ng of purified NalC-his was incubated with 40 ng of C) the annealed oligonucleotide NalC-1 and its reverse complement (corresponds to sequence delineated by endpoints III in Fig. 4A), D) the annealed oligonucleotide NalC-2 and its reverse complement (corresponds to sequence delineated by endpoints IV in Fig. 4A), and E) annealed oligonucleotide NalC-3 and its reverse complement, in which the AGAACTGT sequence of NalC-1 corresponding to the first half of the inverted repeat highlighted in Fig. 4(A) (shaded sequence overlapping the putative −10 regions for *nalC* and PA3720-*armR*) is changed to TCTTGACA.

**Figure 4:**
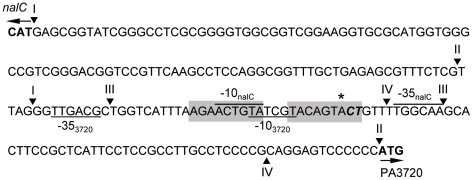
Mapping the PA3720-*armR* transcriptional start site. The *nalC*/PA3720-*armR* intergenic region highlighting the RACE-determined transcription start site for PA3720-*armR* (bolded and italicized), the predicted transcriptional initiation site (marked with an asterisk; assessed using neural network promoter prediction software provided by M. G. Reese at http://www.fruitfly.org/seq_tools/promoter.html), the *nalC* and PA3720 start codons (arrows), and putative −10/−35 sites for *nalC* (overlined) and PA3720-*armR* (underlined) promoters. The end-points of PCR-generated PA3720-distal (I) and -proximal (II) fragments and oligonucleotides (III and IV) used in EMSAs with purified NalC (see Fig. 2B) are identified by arrowheads above the sequence. The shaded sequence corresponds to an inverted repeat and possible NalC-binding site.

In an attempt to localize the NalC binding site NalC binding to ca. 100 bp PA3720-proximal and ca. 100 bp PA3720 distal fragments was assessed, with binding confirmed only for the PA3720-proximal fragment (Fig. 3A, cf Fig. 3B). This fragment (with endpoints II) included putative promoters for *nalC* and PA3720-*armR* as well as an inverted repeat sequence that might serve as the NalC-binding site (Fig. 4). To assess the latter, a 45 bp fragment encompassing the inverted repeat (IR; endpoints III in Fig. 4) was used in an EMSA and NalC binding was observed (Fig. 3C). An identically-sized fragment lacking the IR and encompassing a region more PA3720-proximal (endpoints IV in Fig. 4) failed to bind NalC (Fig. 3D) and little if any binding was observed to a fragment encompassing the repeat region (endpoints III) in which the repeat sequence was mutated, and then only at the highest concentration of NalC (Fig. 3E). This confirmed the repeat region as the NalC-binding site. Interestingly, a 21-bp fragment carrying only the IR failed to bind NalC (data not shown) indicating that the proper disposition of the repeat region for NalC binding required flanking sequence. The IR-containing region to which NalC bound contains the putative promoter regions for *nalC* and PA3720-*armR* [predicted using neural network promoter prediction (http://www.fruitfly.org/seq_tools/promoter.html)] (Fig. 4) as would be expected. Using 5′-RACE the PA3720-*armR* transcription start site was mapped to an adjacent CT pair of bases (exact base could not be determined unequivocally owing to limitations of the RACE), which occurred downstream of the putative PA3720-*armR* -10 region and immediately downstream of the predicted (using neural network) start site (Fig. 4). This is consistent with NalC regulating expression of the PA3720-*armR* operon.

### PCP induction of *mexAB-oprM* is NalC and MexR-dependent but PA3720-ArmR-independent

**Figure 5:**
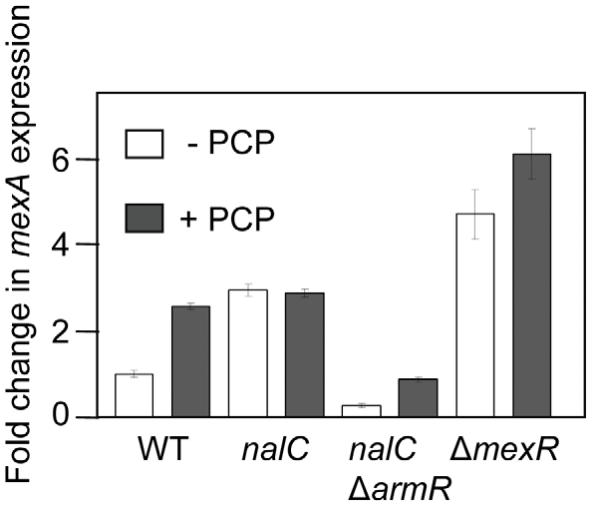
NalC and MexR dependence of PCP induction of *mexAB-oprM*. Expression of *mexA* (as a measure of *mexAB-oprM*) was assessed in *P. aeruginosa* strains K767 (wild type; WT), K1454 (*nalC*), K2276 (*nalC* Δ*armR*) and K2568 (Δ*mexR*) exposed or not to PCP (0.75 mM; 1.5 hr) using qRT-PCR. Expression was normalized to *rpsL* controls and is reported relative (fold change) to untreated *P. aeruginosa* K767. Results shown are the mean +/− standard error of one cDNA sample for each, processed in triplicate, and are representative of 2 independent experiments.

**Figure 6:**
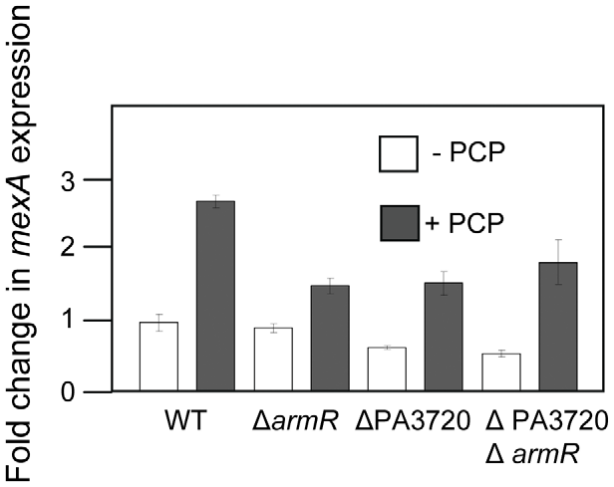
ArmR-/PA3720-independence of PCP induction of *mexAB-oprM*. Expression of *mexA* (as a measure of *mexAB-oprM*) was assessed in *P. aeruginosa* strains K767 (wild type; WT), K3145 (Δ*armR*), K3146 (ΔPA3720) and K3130 (ΔPA3720-*armR*) exposed or not to PCP (0.75 mM; 1.5 hr) using qRT-PCR. Expression was normalized to *rpsL* controls and is reported relative (fold change) to untreated *P. aeruginosa* K767. Results shown are the mean +/− standard error of one cDNA sample for each, processed in triplicate, and are representative of 2 independent experiments.

In addition to its induction of PA320-*armR*, PCP was previously shown to induce the MexR-regulated *mexAB-oprM* multidrug efflux operon [45], results that have been confirmed here (modest ca. 3-fold induction; Fig. 5). Mutations in *nalC* and *mexR* yielded elevated expression of *mexAB-oprM* (*nalC*, 3-fold; *mexR*, 5-fold; Fig. 5), as seen previously [45], and this was no longer responsive to PCP (Fig. 5). Thus, PCP operates though these regulators in promoting *mexAB-oprM* expression. Given that NalC controls expression of the ArmR anti-repressor that modulates MexR repression of *mexAB-oprM*, the obvious interpretation of these results is that PCP as a NalC effector affords PA3720-*armR* expression, with ArmR modulating MexR activity to effect *mexAB-oprM* expression. To assess this, PCP induction of the efflux operon was examined in *armR* mutant strain K3145. As seen in Fig. 6, *mexAB-oprM* was still induced in response to PCP treatment, albeit to a lesser extent, indicating that PCP induction of this efflux operon can occur independently of its promotion of ArmR expression. PA3720 encodes a protein of unknown function that, like *armR*, is NalC regulated [37] and PCP inducible [45] (Fig. 1) and which might, therefore, play a role in PCP induction of *mexAB-oprM*. Thus, PCP induction of *mexAB-oprM* was assessed in a mutant, K3146, lacking PA3720. Again, however, PCP induction of *mexAB-oprM* was retained in the mutant (Fig. 6). Similarly, PCP induction of *mexAB-oprM* was seen in a mutant strain, K3130, lacking both PA3720 and *armR* (Fig. 6), ruling out both genes independently mediating PCP induction of the efflux operon. Similarly, while loss of *armR* compromised the *mexAB-oprM* overexpression seen in a *nalC* mutant (Fig. 5) in agreement with previous results [37], PCP induction of *mexAB-oprM* was retained in the *nalC* Δ*armR* mutant K2276 (Fig. 5) again indicating that PCP induction of the efflux operon can occur independently of ArmR. Interestingly, however, and in contrast to what was observed when *armR* was eliminated in wild type strain K767, where there was little or no impact on basal levels of *mexAB-oprM* expression (in the absence of PCP) (Fig. 6), loss of *armR* in a *nalC* background reduced expression of *mexAB-oprM* to below the basal levels seen in K767 (Fig. 5).

### PCP is not a MexR effector

**Figure 7:**
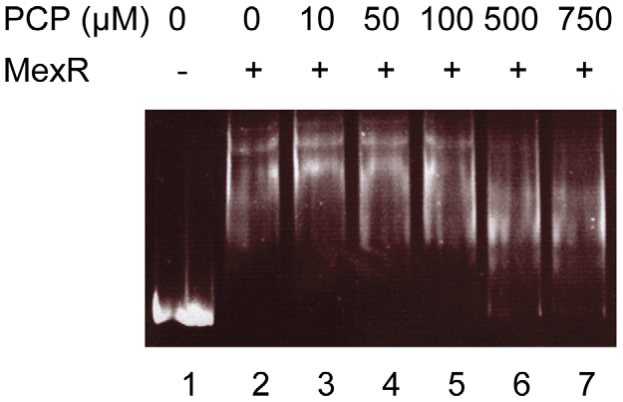
Lack of impact of PCP on MexR repressor binding to the *mexR-mexA* intergenic region. Mobility shift assay in which purified MexR-His (500 ng) was incubated with 40 ng of a 351-bp DNA fragment encompassing the *mexR-mexA* intergenic region and increasing amounts of PCP as indicated. Lane 1, DNA only control.

The observation that PCP induction of *mexAB-oprM* is dependent on MexR but independent of PA3720-*armR* suggested that PCP may be capable of directly modulating MexR activity to effect efflux gene expression. To assess this, the impact of PCP on MexR binding to a DNA fragment containing the *mexAB-oprM* promoter region was examined using EMSA. As seen in Fig. 7 MexR binding was observed, revealing a complex pattern of DNA binding suggestive of variable occupancy of multiple MexR binding sites and/or cooperative binding of MexR to its target DNA. Inn any case, PCP had no impact on MexR binding at concentrations up to 100 µM, and while there were qualitative differences in the pattern of shifted DNA at the highest concentrations (500 and 750 µM) virtually all of the *mexAB-oprM* promoter-containing DNA remained bound to MexR at these concentrations of PCP. Thus, in contrast to NalC, PCP is not an effector for MexR.

## Discussion

ArmR is an anti-repressor that modulates MexR repressor activity in *nalC* mutants that hyperexpress the PA3720-*armR* operon, accounting for the elevated expression of the *mexAB-oprM* multidrug efflux operon in such mutants [37]. In light of the impact of PCP on NalC repression of the PA3720-*armR* operon and the NalC dependence of PCP induction of *mexAB-oprM* it seemed reasonable to suppose that PCP induction of the efflux operon followed from elevated *armR* expression and subsequent ArmR-dependent modulation of MexR repression of *mexAB-oprM*. Indeed, this was the conclusion of a recent study, published while this work was in progress, demonstrating that PCP induction of PA3720-*armR* expression results from PCP directly modulating NalC's repression of this operon [46]. Still, the requirement for ArmR in PCP-inducible *mexAB-oprM* expression was not assessed in that study and results presented here clearly demonstrate that PCP induction of the efflux operon occurs in the absence of the anti-repressor. While there seemed to be some reduction in the absolute levels of PCP-inducible *mexAB-oprM* expression in the absence of *armR*, all mutants lacking *armR* (i.e., K767Δ*armR*, *nalC*Δ*armR*, K767ΔPA3720Δ*armR*) showed fold increases in expression of the efflux genes in the presence of PCP (2- to 3-fold increase) that were similar to wild type strain K767 (ca. 3-fold).

While it is unlikely that elevated *armR* expression in PCP-treated *P. aeruginosa* would not contribute to *mexAB-oprM* expression as a result of ArmR modulation of MexR repressor activity, these results do indicate that there are other mechanisms by which PCP can promote *mexAB-oprM* expression, probably in parallel with the ArmR-dependent mechanism. Such a mechanism appears not to involve PA3720, whose function still remains to be elucidated, but does require MexR, which may respond to PCP. Given the observation that PCP does not impact MexR binding to the *mexAB-oprM* promoter containing DNA *in vitro*, one possibility is that *P. aeruginosa* exposure to PCP promotes *in vivo* production of MexR effector molecule(s) that facilitate *mexAB-oprM* derepression. In this vein, it has been shown that MexR binding to the *mexAB-oprM* promoter region and its control of efflux gene expression is impacted by oxidative stress, MexR being a redox responsive regulator whose ability to bind and repress *mexAB-oprM* is compromised when the protein is oxidized [33], [34]. Thus, exposure to hydroperoxide oxidizes MexR and alleviates promoter binding *in vitro* and induces *mexAB-oprM* expression *in vivo* [34]. One possibility, then, is that PCP exposure generates some form of oxidative stress in *P. aeruginosa*. Intriguingly, PCP has been shown to dramatically increase O_2_ flux in *P. aeruginosa*, generating an oxidative stress that could impact MexR activity [54]. Puzzlingly, PCP induction of *mexAB-oprM* is absent in *nalC* mutant K1454, despite the fact that efflux gene expression is less than what is seen in a *mexR* null strain (K2568) – while much of MexR is inactive as a repressor in K1454, being bound by ArmR, clearly some MexR repression of *mexAB-oprM* is still occurring and would, presumably, be susceptible to modulation by PCP (or PCP-generated effectors). Still, there is some unexplained complexity to *mexAB-oprM* expression in a *nalC* strain given the greater impact of *armR* loss on efflux gene expression in a *nalC* background as compared to an otherwise wild type background where in absolute terms *mexAB-oprM* expression is less (2-fold; Fig. 5) in a *nalC* Δ*armR* mutant (K2276) than a Δ*armR* mutant (K3145). Whether this explains the anomaly, there is likely to be differences in the expression of at least some genes in K2276 vs. K3145, with PA3720 presumably up-regulated in the former but not the latter and any other genes impacted by loss of *nalC* and/or upregulation of PA3720 differentially expressed in the two strains. How these might influence *mexAB-oprM* expression is uncertain.

The modulation of NalC repressor activity by PCP (and other chlorinated phenols [46]) occurs at high levels (high µM to low mM) for an effector molecule suggesting that it only mimics an intended NalC effector and ultimate inducer of *mexAB-oprM*, likely a phenolic of some kind. Plants are common sources of phenolic compounds, many of which have antimicrobial activity [55]–[58], and these may be the intended inducers/substrates for MexAB-OprM. While attempts were made to assess the ability of plant extracts to ‘interact’ with NalC and to induce *mexAB-oprM* expression technical issues (extract insolubility in aqueous solutions) compromised these efforts.

## References

[1] Kerr KG, Snelling AM (2009). *Pseudomonas aeruginosa*: a formidable and ever-present adversary.. J Hosp Infect.

[2] Poole K (2011). *Pseudomonas aeruginosa*: resistance to the max.. Front Microbio.

[3] Poole K (2005). Efflux-mediated antimicrobial resistance.. J Antimicrob Chemother.

[4] Poole K (2007). Efflux pumps as antimicrobial resistance mechanisms.. Ann Med.

[5] Masuda N, Sakagawa E, Ohya S, Gotoh N, Tsujimoto H (2000). Substrate specificities of MexAB-OprM, MexCD-OprJ, and MexXY-OprM efflux pumps in *Pseudomonas aeruginosa*.. Antimicrob Agents Chemother.

[6] Chuanchuen R, Beinlich K, Hoang TT, Becher A, Karkhoff-Schweizer RR (2001). Cross-resistance between triclosan and antibiotics in *Pseudomonas aeruginosa* is mediated by multidrug efflux pumps: exposure of a susceptible mutant strain to triclosan selects *nfxB* mutants overexpressing MexCD-OprJ.. Antimicrob Agents Chemother.

[7] Li X-Z, Zhang L, Poole K (1998). Role of the multidrug efflux systems of *Pseudomonas aeruginosa* in organic solvent tolerance.. J Bacteriol.

[8] Li XZ, Poole K (1999). Organic solvent-tolerant mutants of *Pseudomonas aeruginosa* display multiple antibiotic resistance.. Can J Microbiol.

[9] Srikumar R, Li X-Z, Poole K (1997). Inner membrane efflux components are responsible for the ß-lactam specificity of multidrug efflux pumps in *Pseudomonas aeruginosa*.. J Bacteriol.

[10] Srikumar R, Kon T, Gotoh N, Poole K (1998). Expression of *Pseudomonas aeruginosa* multidrug efflux pumps MexA-MexB-OprM and MexC-MexD-OprJ in a multidrug-sensitive *Escherichia coli* strain.. Antimicrob Agents Chemother.

[11] Srikumar R, Poole K (1999). Demonstration of ethidium bromide efflux by multiresistant pumps of *Pseudomonas aeruginosa*.. Clin Microbiol Infect.

[12] Schweizer HP (1998). Intrinsic resistance to inhibitors of fatty acid biosynthesis in *Pseudomonas aeruginosa* is due to efflux: application of a novel technique for generation of unmarked chromosomal mutations for the study of efflux systems.. Antimicrob Agents Chemother.

[13] Evans K, Passador L, Srikumar R, Tsang E, Nezezon J (1998). Influence of the MexAB-OprM multidrug efflux system on quorum-sensing in *Pseudmonas aeruginosa*.. J Bacteriol.

[14] Pearson JP, Van Delden C, Iglewski BH (1999). Active efflux and diffusion are involved in transport of *Pseudomonas aeruginosa* cell-to-cell signals.. J Bacteriol.

[15] Poole K (2000). Efflux-mediated resistance to fluoroquinolones in Gram-negative bacteria.. Antimicrob Agents Chemother.

[16] Henrichfreise B, Wiegand I, Pfister W, Wiedemann B (2007). Resistance mechanisms of multiresistant *Pseudomonas aeruginosa* strains from Germany and correlation with hypermutation.. Antimicrob Agents Chemother.

[17] Dupont P, Hocquet D, Jeannot K, Chavanet P, Plesiat P (2005). Bacteriostatic and bactericidal activities of eight fluoroquinolones against MexAB-OprM-overproducing clinical strains of *Pseudomonas aeruginosa*.. J Antimicrob Chemother.

[18] Cavallo JD, Hocquet D, Plesiat P, Fabre R, Roussel-Delvallez M (2007). Susceptibility of *Pseudomonas aeruginosa* to antimicrobials: a 2004 French multicentre hospital study.. J Antimicrob Chemother.

[19] Hocquet D, Roussel-Delvallez M, Cavallo JD, Plesiat P (2007). MexAB-OprM- and MexXY-overproducing mutants are very prevalent among clinical strains of *Pseudomonas aeruginosa* with reduced susceptibility to ticarcillin.. Antimicrob Agents Chemother.

[20] Boutoille D, Corvec S, Caroff N, Giraudeau C, Espaze E (2004). Detection of an IS*21* insertion sequence in the *mexR* gene of *Pseudomonas aeruginosa* increasing ß-lactam resistance.. FEMS Microbiol Lett.

[21] Cavallo JD, Plesiat P, Couetdic G, Leblanc F, Fabre R (2002). Mechanisms of ß-lactam resistance in *Pseudomonas aeruginosa*: prevalence of OprM-overproducing strains in a French multicentre study (1997).. J Antimicrob Chemother.

[22] Wang J, Zhou JY, Qu TT, Shen P, Wei ZQ (2010). Molecular epidemiology and mechanisms of carbapenem resistance in *Pseudomonas aeruginosa* isolates from Chinese hospitals.. Int J Antimicrob Agents.

[23] Pournaras S, Maniati M, Spanakis N, Ikonomidis A, Tassios PT (2005). Spread of efflux pump-overexpressing, non-metallo-ß-lactamase-producing, meropenem-resistant but ceftazidime-susceptible *Pseudomonas aeruginosa* in a region with *bla*
_VIM_ endemicity.. J Antimicrob Chemother.

[24] Srikumar R, Paul CJ, Poole K (2000). Influence of mutations in the *mexR* repressor gene on expression of the MexA-MexB-OprM multidrug efflux system of *Pseudomonas aeruginosa*.. J Bacteriol.

[25] Saito K, Yoneyama H, Nakae T (1999). *nalB*-type mutations causing the overexpression of the MexAB-OprM efflux pump are located in the *mexR* gene of the *Pseudomonas aeruginosa* chromosome.. FEMS Microbiol Lett.

[26] Ziha-Zarifi I, Llanes C, Koehler T, Pechere J-C, Plesiat P (1999). In vivo emergence of multidrug-resistant mutants of *Pseudomonas aeruginosa* overexpressing the active efflux system MexA-MexB-OprM.. Antimicrob Agents Chemother.

[27] Jalal S, Wretlind B (1998). Mechanisms of quinolone resistance in clinical strains of *Pseudomonas aeruginosa*.. Microbiol Drug Resist.

[28] Jalal S, Ciofu O, Hoiby N, Gotoh N, Wretlind B (2000). Molecular mechanisms of fluoroquinolone resistance in *Pseudomonas aeruginosa* isolates from cystic fibrosis.. Antimicrob Agents Chemother.

[29] Evans K, Adewoye L, Poole K (2001). MexR repressor of the *mexAB-oprM* multidrug efflux operon of *Pseudomonas aeruginosa*: identification of MexR binding sites in the *mexA-mexR* intergenic region.. J Bacteriol.

[30] Morita Y, Cao L, Gould G, Avison MB, Poole K (2006). *nalD* encodes a second repressor of the *mexAB-oprM* multidrug efflux operon of *Pseudomonas aeruginosa*.. J Bacteriol.

[31] Lim D, Poole K, Strynadka NC (2002). Crystal structure of the MexR repressor from the multidrug efflux operon in *Pseudomonas aeruginosa*.. J Biol Chem.

[32] Sanchez P, Rojo F, Martinez JL (2002). Transcriptional regulation of *mexR*, the repressor of *Pseudomonas aeruginosa mexAB-oprM* multidrug efflux pump.. FEMS Microbiol Lett.

[33] Chen H, Yi C, Zhang J, Zhang W, Ge Z (2010). Structural insight into the oxidation-sensing mechanism of the antibiotic resistance of regulator MexR.. EMBO Rep.

[34] Chen H, Hu J, Chen PR, Lan L, Li Z (2008). The *Pseudomonas aeruginosa* multidrug efflux regulator MexR uses an oxidation-sensing mechanism.. Proc Natl Acad Sci U S A.

[35] Daigle DM, Cao L, Fraud S, Wilke MS, Pacey A (2007). Protein modulator of multidrug efflux gene expression in *Pseudomonas aeruginosa*.. J Bacteriol.

[36] Wilke MS, Heller M, Creagh AL, Haynes CA, McIntosh LP (2008). The crystal structure of MexR from *Pseudomonas aeruginosa* in complex with its antirepressor ArmR.. Proc Natl Acad Sci U S A.

[37] Cao L, Srikumar R, Poole K (2004). MexAB-OprM hyperexpression in NalC type multidrug resistant *Pseudomonas aeruginosa*: identification and characterization of the *nalC* gene encoding a repressor of PA3720-PA3719.. Mol Microbiol.

[38] Llanes C, Hocquet D, Vogne C, Benali-Baitich D, Neuwirth C (2004). Clinical strains of *Pseudomonas aeruginosa* overproducing MexAB-OprM and MexXY efflux pumps simultaneously.. Antimicrob Agents Chemother.

[39] Sobel ML, Hocquet D, Cao L, Plesiat P, Poole K (2005). Mutations in PA3574 (*nalD*) lead to increased MexAB-OprM expression and multidrug resistance in lab and clinical isolates of *Pseudomonas aeruginosa*.. Antimicrob Agents Chemother.

[40] Fraud S, Campigotto AJ, Chen Z, Poole K (2008). The MexCD-OprJ multidrug efflux system of *Pseudomonas aeruginosa*: involvement in chlorhexidine resistance and induction by membrane damaging agents dependent upon the AlgU stress-response sigma factor.. Antimicrob Agents Chemother.

[41] Fetar H, Gilmour C, Klinoski R, Daigle DM, Dean CR (2011). *mexEF-oprN* multidrug efflux operon of *Pseudomonas aeruginosa*: regulation by the MexT activator in response to nitrosative stress and chloramphenicol.. Antimicrob Agents Chemother.

[42] Fraud S, Poole K (2010). Oxidative stress induction of the *mexXY* multidrug efflux genes and promotion of aminoglycoside resistance development in *Pseudomonas aeruginosa*.. Antimicrob Agents Chemother.

[43] Jeannot K, Sobel ML, El Garch F, Poole K, Plesiat P (2005). Induction of the MexXY efflux pump in *Pseudomonas aeruginosa* is dependent on drug-ribosome interaction.. J Bacteriol.

[44] Morita Y, Gilmour C, Metcalf D, Poole K (2009). Translational control of the antibiotic inducibility of the PA5471 gene required for *mexXY* multidrug efflux gene expression in *Pseudomonas aeruginosa*.. J Bacteriol.

[45] Muller JF, Stevens AM, Craig J, Love NG (2007). Transcriptome analysis reveals that multidrug efflux genes are upregulated to protect *Pseudomonas aeruginosa* from pentachlorophenol stress.. Appl Environ Microbiol.

[46] Ghosh S, Cremers CM, Jakob U, Love NG (2011). Chlorinated phenols control the expression of the multi-drug resistance efflux pump MexAB-OprM in *Pseudomonas aeruginosa* by activating NalC.. Mol Microbiol.

[47] Morita Y, Sobel ML, Poole K (2006). Antibiotic inducibility of the MexXY multidrug efflux system of *Pseudomonas aeruginosa*: involvement of the antibiotic-inducible PA5471 gene product.. J Bacteriol.

[48] Sobel ML, McKay GA, Poole K (2003). Contribution of the MexXY multidrug transporter to aminoglycoside resistance in *Pseudomonas aeruginosa* clinical isolates.. Antimicrob Agents Chemother.

[49] Sheu DS, Wang YT, Lee CY (2000). Rapid detection of polyhydroxyalkanoate-accumulating bacteria isolated from the environment by colony PCR.. Microbiology.

[50] Sambrook J, Russell DW (2001). Molecular cloning: a laboratory manual, 3rd ed.

[51] Choi KH, Kumar A, Schweizer HP (2005). A 10-min method for preparation of highly electrocompetent *Pseudomonas aeruginosa* cells: Application for DNA fragment transfer between chromosomes and plasmid transformation.. J Microbiol Methods.

[52] Jo JT, Brinkman FS, Hancock RE (2003). Aminoglycoside efflux in *Pseudomonas aeruginosa*: involvement of novel outer membrane proteins.. Antimicrob Agents Chemother.

[53] Frohman MA (1993). Rapid amplification of complementary DNA ends for generation of full-length complementary DNAs: thermal RACE.. Methods Enzymol.

[54] McLamore ES, Zhang W, Porterfield DM, Banks MK (2010). Membrane-aerated biofilm proton and oxygen flux during chemical toxin exposure.. Environ Sci Technol.

[55] Park YJ, Biswas R, Phillips RD, Chen J (2011). Antibacterial activities of blueberry and muscadine phenolic extracts.. J Food Sci.

[56] Zhao J, Lou J, Mou Y, Li P, Wu J (2011). Diterpenoid tanshinones and phenolic acids from cultured hairy roots of *Salvia miltiorrhiza* Bunge and their antimicrobial activities.. Molecules.

[57] Lanoue A, Burlat V, Schurr U, Rose US (2010). Induced root-secreted phenolic compounds as a belowground plant defense.. Plant Signal Behav.

[58] Masoko P, Gololo SS, Mokgotho MP, Eloff JN, Howard RI (2010). Evaluation of the antioxidant, antibacterial, and antiproliferative activities of the acetone extract of the roots of *Senna italica* (Fabaceae).. Afr J Tradit Complement Altern Med.

[59] Ausubel FM, Brent R, Kingston RE, Moore DD, Seidman JG (1992). Short protocols in molecular biology, 2nd ed.

[60] Studier FW (1991). Use of bacteriophage T7 lysozyme to improve an inducible T7 expression system.. J Mol Biol.

[61] Simon R, Priefer U, Puehler A (1983). A broad host range mobilization system for *in vivo* genetic engineering: transposon mutagenesis in Gram-negative bacteria.. Biotechnology.

[62] Miller VL, Mekalanos JJ (1988). A novel suicide vector and its use in construction of insertion mutations: osmoregulation of outer membrane proteins and virulence determinants in *Vibrio cholerae* requires *toxR*.. J Bacteriol.

[63] Masuda N, Ohya S (1992). Cross-resistance to meropenem, cephems, and quinolones in *Pseudomonas aeruginosa*.. Antimicrob Agents Chemother.

[64] Hoang TT, Karkhoff-Schweizer RR, Kutchma AJ, Schweizer HP (1998). A broad-host-range Flp-FRT recombination system for site-specific excision of chromosomally-located DNA sequences: application for isolation of unmarked *Pseudomonas aeruginosa* mutants.. Gene.

